# Health facility-based data on women receiving sulphadoxine-pyrimethamine during pregnancy in Tanzania: lessons to learn from a cross-sectional survey in Mkuranga and Mufindi districts and other national survey reports

**DOI:** 10.1186/1742-4755-11-6

**Published:** 2014-01-16

**Authors:** Godfrey M Mubyazi, Jens Byskov, Pascal Magnussen, Ib C Bygbjerg, Jasper N Ijumba, Mufungo Marero, Leonard EG Mboera, Fabrizio Molteni, Paul Bloch

**Affiliations:** 1 Department of Health Systems & Policy Research & Centre for Enhancement of Effective Malaria Interventions (CEEMI), National Institute for Medical Research (NIMR) - Headquarters, 2448 Ocean Road, P.O. Box 9653, Dar-Es-Salaam, Tanzania; 2 DBL - Centre for Health Research and Development, Faculty of Life Sciences, University of Copenhagen, Copenhagen, Denmark; 3 Institute of International Health, Immunology and Microbiology, Faculty of Health Sciences, University of Copenhagen, Bleddamsvej 3, DK 2200 Copenhagen N, Denmark; 4 The Nelson Mandela African Institute of Science and Technology, P.O. Box 447, Arusha, Tanzania; 5 Ministry of Health and Social Welfare, National Malaria Control Programme (NMCP), Dar Es Salaam, P.O. Box 9083, Dar es Salaam, Tanzania; 6 NIMR, Directorate of Information Technology and Communication, National Institute for Medical Research, Headquarters, P.O. Box 9653, Dar es Salaam, Tanzania; 7 Steno Diabetes Center, Steno Health Promotion Center, Gentofte, Denmark

**Keywords:** Intermittent, Health information, Malaria prevention, Antenatal care

## Abstract

**Background:**

A study of health facility (HF) data on women receiving sulphadoxine-pyrimethamine (SP) for intermittent preventive treatment of malaria during pregnancy (IPTp) was carried out at antenatal care clinics in Mkuranga and Mufindi districts.

**Methods:**

A review of health management information system (HMIS) registers, interviews with health-care workers (HWs) and district and national level malaria control program managers corroborated by inter-temporal assessment through observations at HF levels. Statistical data were analyzed in Excel and interpreted in triangulation with qualitative data from interviews and observations.

**Results:**

Data indicated that IPTp doses administered to women were inadequate and partly inconsistent. HMIS registers lacked space for IPT records, forcing HWs to manipulate their record-keeping. The proportion/number of IPTp recipients in related to the supply of SP for free delivery, to women’s attendance behaviours, showed variation by quarter and year of reporting.

**Conclusion:**

It is impossible to achieve rational health service planning when the HMIS is weak. Whilst it is acknowledged that the HMIS is already overloaded, concerted measures are urgently needed to accommodate data on new interventions and other vertical programs if malaria programs are to achieve their goals.

## Background

There is a common adage that ‘information is power’. This means that correct, adequate and timely information is fundamental for more realistic decisions. According to the World Health organization (WHO), faster and better information helps to improve health-care service planning and delivery, among other things [1]. Generally, the information related to health is not always sufficient if at all accessible everywhere and to everybody in the world. The WHO’s experts in health metrics depict that although most of the low and middle income countries have been using plans with indicators and targets to monitor progress and performance, the availability and quality of data hamper their ability to accurately do so and this true for the general services or activities and for services related to specific programs or initiatives [1]. Lack or shortage of reliable data is a challenge to planners since it contributes to setting targets based on assumptions or speculations and at the end of the day it may be difficult to judge whether or not the targets set were realistic. Critical debates are likely to continue occurring if there is tendency of the data/information reported from different sources or agencies to be overlapping or contradicting to each other. Analysts have observed that the debate on health information systems has generally been abound mainly on the different forums regarding which data source is preferable for developing and tracking health system targets, documenting best practices or effectiveness of interventions, and identifying gaps in performance. In this regard, there are observers who argue that households and health facility (HF) surveys yield better quality information than self-reported routine health information systems (RHIS) that is commonly known as health management information systems (HMIS). Others perceive the RHIS/HMIS to be costly, producing low quality and mostly irrelevant information, hence contributing less to decision-making processes. Meanwhile, there are those who still observe a missing point in the debate raised so far on HMIS issue by arguing that there is failure of different analysts to recognize that each method of data collection serves a different purpose and has its own strengths and weaknesses [2]. Malaria in pregnancy (MiP) sometimes called malaria during pregnancy or pregnancy attributable malaria is one of the areas in which data deficiencies are reported [3-5], leave alone the debates expressed about the methodological limitations relating to measurement of the clinical and operational effectiveness of this novel intervention strategy performed by different researchers in different studies [6].

For many years, MiP has continued being known to contribute to the regrettable maternal morbidity and mortality rates in sub-Sahara Africa (SSA). The most popular consequences of MiP include anemia, neurological disorders, placental infections that may lead to inter-uterine growth retardations, spontaneous abortion, stillbirth, low birth-weight and child mortality immediately after delivery, and even maternal death [7,8]. Effective control and possibly elimination of MiP continue being a great challenge in SSA despite the reports indicating encouraging progress so far noted in several countries when the control of malaria is done by using a combination of methods. Some of the methods officially recommended and used include the intermittent presumptive/preventive treatment during pregnancy (IPTp) and insecticide-treated nets (ITNs). These methods effectively used by pregnant women and accompanied by adherence to appropriate case management guided by early diagnosis and use of appropriate treatment regimen, have made a significant contribution to MiP control [6,9]. Fighting MiP through the effective administration of sulphurdoxine-pyrimethamine (SP) given to pregnant women attending antenatal care (ANC) clinics as an IPTp strategy is one of the key recommendations of the WHO. Most of the countries located in both the stable and unstable malaria transmission areas have responded by officially implementing this strategy in their ANC systems [7,10]. The focal point of implementation has been the antenatal care (ANC) clinics because that is where many pregnant women can be mobilized for giving this service [4,11].

In Tanzania, the IPTp-SP strategy was officially launched in 2001 [12]. This was right decision since Tanzania is one of the top three countries within SSA whereby malaria is the leading public health problem [13], and one of the countries represented by the delegates at the signing of the Abuja Declaration in 2000. Under the latter Declaration, African heads of states declared that their governments would increase political will by investing in malaria prevention and supporting all initiatives aimed at promotion of effective use of ITNs and IPTp with SP against MiP, focusing on the pregnant women utilizing the available ANC services. The initial national targets for IPTp and ITNs coverage of the pregnant women in line with the Abuja Declaration was: (i) to cover at least 60% of all the registered ANC clients whereby each client would receive at least two doses of SP for IPTp; (ii) ensuring that same target of the ANC clients was achieved for the same category of the women who were sleeping under ITNs. The target year of attaining this goal was 2005 [14]. Under the Roll Back Malaria (RBM) Initiative, some countries decided to revise the ITNs and IPTp coverage rates. The new rates for both IPTp and ITNs were increased at least 80% by 2010 [4,15]. Tanzania has also been one of the supporters of the Millennium Development Goals (MDGs) 4 and 5 set in 2000 with the aim of improving the indicators for maternal and child health by 2015 [16]. Unfortunately, the evidence from a number of evaluations in SSA indicates that most of the countries could not reach the initially set Abuja target by 2005 [5,7]. The reports given, however, show that there have been variations within and between the countries evaluated in the countries’ achievements. These variations are linked to, among other things, the deficiencies observed in terms of system of recording and reporting of the data including data for IPTp-SP and ANC attendance in general, and that this has remained one of the challenges facing national malaria control programmes (NMCP) [15,17,18]. The deficiencies or weaknesses observed in data management systems are reported to be contributed by numerous factors. In several country cases it has been noted that the implementation of the IPTp strategy took place before preparations were adequately done to ensure adequate training/orientation of frontline health workers (HWs). This is one of the reasons for the observed low rates of coverage of the ANC clients with IPTp doses. Meanwhile, other evidence from selected countries indicates that the introduction of training programmes to frontline HWs led to dramatic improvements in IPTp administration [5,19].

The Tanzanian Government’s decision to introduce IPTp strategy in the health care system came as a replacement of chloroquine as a weekly prophylaxis [20] with SP. The guidelines require the frontline HWs to administer SP to eligible women under the supervision system popular directly observed therapy (DOT) while the women concerned are still at the ANC clinics as recommended by WHO [11,15]. However, still the same Tanzania national guidelines recommended the first of dose of SP to be given between the 20^th^ and 24^th^ week of pregnancy during the second trimester while the second one to be administered during the third trimester, but not later than the 36^th^ week, and particularly between the 28^th^ and 32^nd^ weeks [21]. Although this guideline of the timing of IPTp doses seemed to confuse some of the frontline HWs [18], the newly updated guidelines given by the WHO in 2012 allow more than two doses to be delivered, subject to specified conditions [11]. Therefore, the national guidelines of Tanzania would be reviewed and frontline service givers more informed and sensitized to comply with [NMCP Officers, per comm.].

To put the present paper in the perspective for its readers to be able to realize the rationale behind its publication, the following additional account on the national health management information system (HMIS) refers: IPTp-SP as one of national malaria control strategies in Tanzania was introduced before the Ministry of Health and Social Welfare (MoHSW) decided to revise its HMIS. The revision was aimed at creating new data registers that would allow the routine patient data system accommodate the IPTp data, along with the data representing the pregnant women receiving the National ITN Strategy (NATNETs)’s discounted vouchers, among other health service packages delivered to pregnant women and other population categories. Unfortunately, the need for keeping data for different services in the same old registers has practically caused difficulties or inconvenience to the frontline HWs who are responsible for documenting records relating to all the services they have delivered at health facility (HF) level on specific official days. The challenge has also been facing the district Council Health Management Team (CHMT) members responsible for using the gathered HF-based data usually recorded in the HMIS registers/books for official purposes. These include planning for various health service related activities [18,22,23]. The national HMIS (abbreviated as MTUHA in Kiswahili who is full wording is *Mfumo wa Taarifa za Uendeshaji wa Huduma za Afya*) was introduced many years ago [24], and actually before the introduction of the IPTp-SP strategy in 2001. Following its official introduction, the MTUHA registers continued being noted of lacking designated spaces for accommodating data/records for the services delivered under the newly introduced interventions. Data relating to IPTp-SP against MiP and those relating to the NATNETs Discount Voucher Scheme [18] were among other categories of the data that caused problems of accommodation in the current MTUHA registers. This has resulted into more problems at lower HF (e.g. dispensary) levels that have always been characterized as mostly facing chronic human resource shortages due to understaffing and lack of supportive supervision on many issues including those relating to data/record keeping and at times inadequate registers. The frontline HWs stationed at such poorly equipped HFs as in most other peripheral HFs have been faced with the problem of late or no reception of feedback on issues relating to use of the data collected at HF level and a proper system for analyzing such routinely collected data [18,23,25].

The authorities concerned at district and national levels in Tanzania, including the district CHMTs and NMCP under the MoHSW have also been frustrated with implementation of the HMIS. This is mainly due to the seemingly to be frontline HWs’ failure to take records, document such records and report them for the health services delivered in proper manner and/or in time. This has indeed become a serious challenge when it comes to mobilizing the data required for guiding the health service planning and monitoring authorities at district and national levels [18,26]. This problem has been reported to commonly face many developing countries within and outside Africa [27]. Therefore, the WHO has continued advising its member countries to ensure that they have properly functioning HMIS since having the accurately collected, analyzed and stored data could allow informed and rational policy decisions in the health sector to be made [1]. Thus, a proper planning for IPTp services is impossible in the absence of reliable sources of routine health service data mostly collected from HFs, and this is true for the general service data [1,2,28-30] and data for specific interventions like IPTp [18].

The present paper builds on the above background to present and discuss the findings from a study that was undertaken to assess, among other things, the economic and other contextual determinants of acceptability and practicability of IPTp against malaria in Tanzania [31]. Based on the service providers’ and health managers’ viewpoint, the issue of acceptability of IPTp covered under the study were related to such things as whether the strategy was perceived appropriate theoretically/conceptually, and whether it was practicable when it comes to implementation [18,22]. The paper pays attention to the discussion on the data collected from HFs as a representation of the number of pregnant women who had received IPTp-SP doses at ANC levels in rural Tanzanian districts, focusing on Mkuranga and Mufindi districts case study. Attention has been paid to examining the frontline HWs’ compliance with the administration of IPTp doses required by the national focused ANC guidelines, and the issue of proper record-taking in the MTUHA/HMIS registers/books as well as on the pregnant women’s ANC cards. The HF data were taken directly from survey in the study districts, but to strengthen the discussion on such type of the data, additional data were gathered from other (i.e. secondary) sources. These could be used to supplement or challenge the primary data collected, and they include data reviewed from other national or selected districts surveys as part of the status of NMCP’s evaluation of IPTp implementation and coverage. Moreover, qualitative information was obtained through interviews with HWs, district officers and national level managers [18,22]. Additional inputs were obtained from the ANC users and service providers [31,32].

## Methods

### Study design and areas

A large cross-sectional survey was designed in 2005 and undertaken in two districts between November 2005 and February 2007, and the districts concerned were Mkuranga and Mufindi. These districts are found in different regions, Mkuranga being along the coast and Mufindi in the southern highlands regions. This was part of a PhD research program of the first author in this paper, so had a short time scale for data collection. A large part of the data for this HMIS component from the main study was obtained between March and October 2006 in two the districts. This was done two years after the national focused ANC guidelines were introduced [21]. More details on the selection of the study districts, HFs, and the study population’s socio-economic and health system characteristics are documented elsewhere [32,33]. As originally planned, more up-to-date data collected from the two districts was important for comparison with the data collected from other places in the country through, for example, the zonal, national and even smaller scale (mainly operational research based) surveys [34,35].

### Sampling of health facilities

Selection of the ANC clinics followed a combination of randomization and convenience sampling techniques. The randomization approach was applied in relation to identification of the dispensaries in both districts and health centers (HCs) in Mufindi district only which had five HCs in total out of which only three were picked; in Mkuranga there were only three health centers and both were public or government as popularly known [32,33]. Thus, sampling process initially targeted at the design of the study to cover a mixture of public HFs and private HFs with ANC clinics i.e. where ANC services were being delivered. A convenience sampling approach was adopted where the number of the HFs available in the district concerned was too small to allow randomization to be performed, and this applies for the hospitals covered (2 in Mufindi, 1 in Mkuranga) and the HCs covered in Mkuranga. In summary, 13 HFs and 35 HWs in Mkuranga district and 15 HFs and 43 HWs in Mufindi district were covered until the end of the study. Further details on these issues reported elsewhere [31,33]. At the district level, members of district CHMT were approached considering their role as district health service planners and managers, and in total, seven and eight members were approached in Mkuranga and Mufindi districts respectively [22].

### Data collection techniques

#### Quantitative data

An observational checklist organized in form of a catalogue was used to guide the data collectors in the review of the MTUHA registers at selected HFs. The data on the IPTp doses delivered to the individual ANC clients were collated from routine MTUHA registers at HF level, and partly observations made to cross-check some from a few ANC cards. This activity was performed between March and October 2006, but was followed by additional interviews with HWs and CHMT members between January and February 2007. Data related to the number of women who received IPTp doses each month were found mainly in the MTUHA Book 6 version 2.0, and the main interest was to determine the number (and later on the) proportion of the pregnant women who had received the first dose of SP for IPTp (popular as IPTp-1) and those who received at least the second dose (known as IPTp-2). Flexibility was allowed to collect the data for those who received even more than two doses (e.g. IPTp-3), bearing in mind that it was not a common practice of most of the frontline HWs to administer this dose since the national focused ANC guidelines put emphasis on at least two doses during a woman’s pregnancy period [18,21].

#### Inter-temporal observations

As mentioned above, inter-temporal observations were also done on how the HWs at the visited ANC level behaved when administering SP for IPTp under DOT and recording the client’s data into the MTUHA registers. This helped to establish whether and how the HWs delivering the services complied with DOT system for administering IPTp doses as per guidelines, and the, same guidelines requiring proper record keeping in MTUHA. Observations were made in attempt to cross-check or confirm some of the records shown on a few clients’ ANC cards randomly identified.

#### Qualitative data

The qualitative data were collected via interviews with key informants (in this case, these were the frontline HWs and district level health managers). These two groups of respondents were asked to give their opinions about the strengths (usefulness) and weaknesses (shortcomings) of the MTUHA system of data keeping in relation to IPTp doses administered to pregnant women at the ANC clinic level, and applicability of such data for service planning at district and national levels and for implementation at HF level where they are being collected. In short, the interviews with the key informants specifically related to the context around the quantitative data and perceptions regarding the HMIS/MTUHA reporting of IPTp. To validate some of the reports given by the frontline HWs, confirmatory information was sought from the women who received IPTp doses before by reviewing their ANC cards at the MCH clinics. This approach is consistent to methods applied elsewhere in Tanzania [34,36] and as previously recommended by other experts [37].

Part of the qualitative data was not collected at the same time as the quantitative data were being collected. The HMIS review was performed with reference to the data period (2003 and 2006) while additional interviews were performed in 2007 and later, thus possible recall bias could not be avoided as it is discussed later in the present paper.

### Data analysis

#### Quantitative data

The statistical data were processed for analysis using MS Excel software. Cumulative data for each year and the proportions of IPT dose recipients on monthly (then quarterly) bases were categorized to capture a picture on variations in women’s registered to have taken IPTp doses which could be explained by feedback obtained from interviews with HWs and higher authorities at district and national levels. The data collected from different health facilities were not sufficient for a substantial comparative analysis to reflect the performance of HWs at different levels and types of HFs. Also, the number of the HFs belonging to public authorities and those owned by the private not for profit (i.e. faith-based) organizations (FBO) or private for profit (i.e. commercial) organizations was not the same [30-33]. This made it difficult to undertake a realistic comparison, and that is why based on these shortcomings, the decision made at the analysis stage was to treat the data for each district separately.

Data from several national or zonal surveys collected and compiled by other evaluators are also presented. Based on these, a reflection on the general picture on IPTp delivery and record keeping status in the country could be obtained and compared with the data collected from the HMIS registers at the present study HFs selected in Mkuranga and Mufindi districts.

#### Qualitative data

The qualitative data from field observations and interviews were analyzed manually - no software was used. Using a qualitative content analysis approach as reported elsewhere [32], the study themes were organized in accordance with the study objective(s) and the contents coming out from the study. The contents were taken as far as they could support arguments to be made based on the aspects intended to be covered in attempt to answer the questions in hand, including their ability to provide possible explanation on the statistical (quantitative) data.

#### Data presentation and interpretation

Some of the statistical data were presented in tabular form, others in graphical form, depending on the nature of the data tables (Tables 1 and 2). Looking at the primary data collected from the HFs under the present study, one may start getting a feeling of associating the monthly variations the IPTp-SP delivery to the ANC clients or variations in the ANC attendance behaviors of the pregnant women eligible for receiving IPTp. The other possibility is the issue of SP stock-out at HF levels, but this has been explained or argued in detail under the results section and partly under the discussion section. Thus, the IPTp doses varying in some months indicated that the ANC attendances for the eligible women were presumably higher or lower than the other months. However, this was left to be confirmed by the reports from the key informants and even the HMIS registers during the data collection process. Examining all these, was found to be important in account of the fact that the observed differences might influence/affect the planning for service delivery. This is what was conceptually thought of at the stage of the study design.

**Table 1:** Number of pregnant women registered to have received IPTp-SP doses at selected HFs in Mkuranga district in each quarter of years 2004–2006 (N = 10 HFs for years 2004–2005) and (N = 9 HFs for year 2006)

**Month**	**Year 2004**		**Year 2005**		**Year 2006**	
	**No. of clients who received IPTp-1**	**No. of clients who received IPTp-2**	**No. of clients who received IPTp-1**	**No. of clients who received IPTp-2**	**No. of clients who received IPTp-1**	**No. of clients who received IPTp-2**
*Jan – March*	590	366	946	517	786	512
*April – June*	590	436	963	474	894	474
*July – Sept*	591	373	911	438	807	447
*Oct – Dec.*	618	252	746	238	707	238

**Table 2:** Number of pregnant women registered to have received IPTp-SP doses during each quarter of years 2003–2005 at 14 HFs in Mufindi district, Tanzania

**Month**	**Year 2003**		**Year 2004**		**Year 2005**	
	**No. of IPTp-1 recipients**	**No. of IPTp-2 recipients**	**No. of IPTp-1 recipients**	**No. of IPTp-2 recipients**	**No. of IPTp-1 recipients**	**No. of IPTp-2 recipients**
*Jan – March*	444	186	1021	372	1118	416
*April – June*	1347	412	1242	684	1224	682
*July – Sept*	1180	918	1299	944	1471	959
*Oct – Dec*	1073	734	1316	902	1078	845

**Figure 1:**
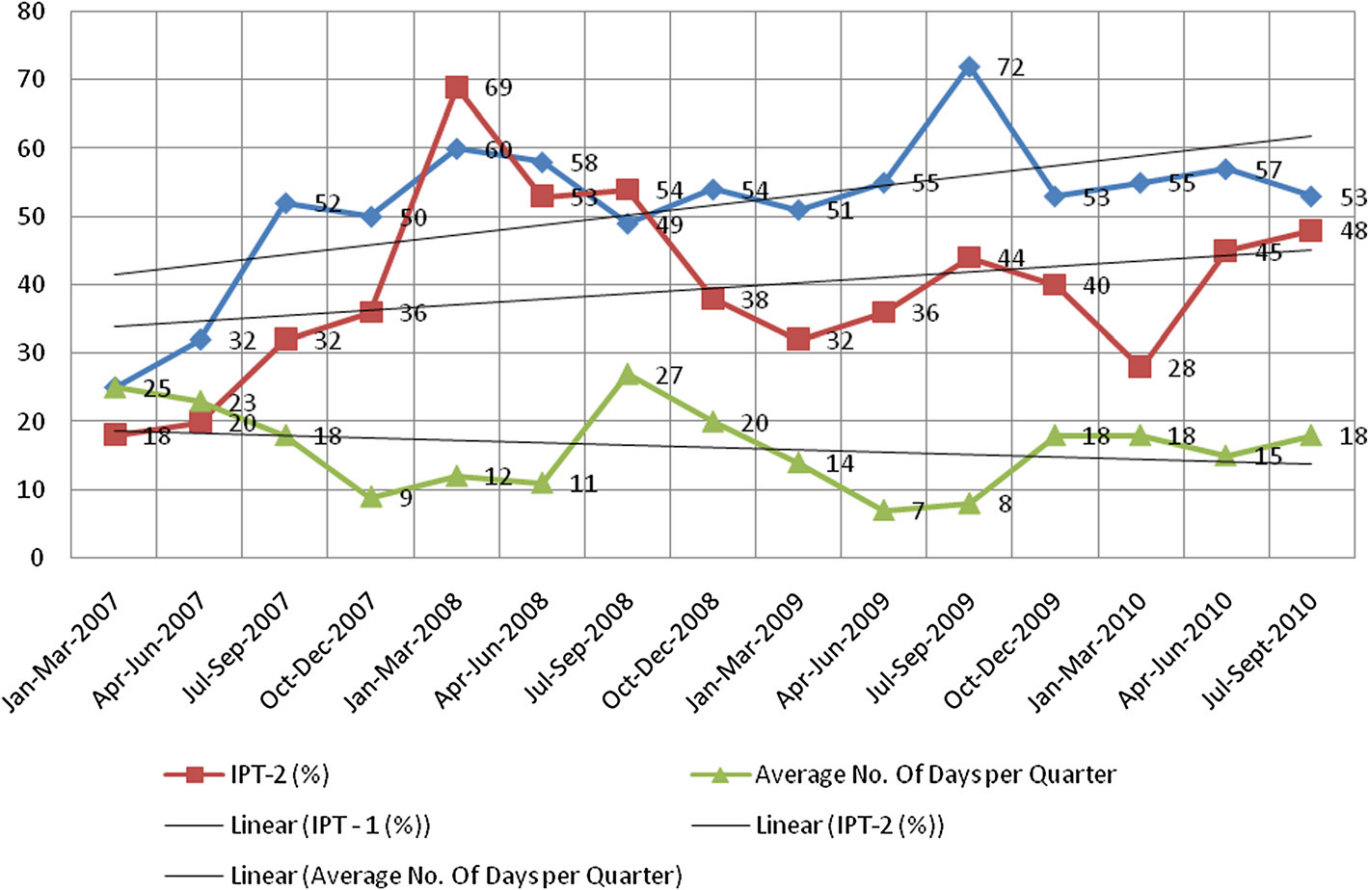
**Rates of SP stockout, IPT- 1 & 2 for pregnant women between January 2007- Sept 2010 (Source: USAID funded MAISHA focused ANC Service Program).***Key*: (i) The abbreviations IPT-1 and IPT-2 as used in this figure are same as IPTp-1 and IPTp-2 respectively used throughout the paper. (ii) The topmost line with figures for specific quarters as indicated in blue and whose legend has not been shown represents the percentages of IPTp-1 recipients; (iii) The straight lines crossing each graphical line with data/figures represent the average number of days per quarter corresponding to the data shown.

The quarterly data (Tables 1 and 2) are meant to illustrate the quarterly coverage pattern since sometimes planning for particular services is done in account of different quarters of the year that are usually influenced by variations in the timing and sometimes amount of the funds and other HFs resource requirements that allocated by the districts or central government departments concerned. The rationale behind presenting data for different quarters of the year is that local government CHMTs in Tanzania usually undertake monitoring and evaluation (M&E) activities on health services issues in the district and this is done on quarterly basis. The aim is to get a picture on the performance indicators for the health services they previously targeted to achieve in accordance with their comprehensive council health plans. Additional data presented in the rest of the tables (Tables 3 and 4) and graphic (Figure 1) are just for augmenting or validating the primary data collected under the present study as shown in Tables 1 and 2.

**Table 3:** Breakdown indicating proportions of pregnant women covered with IPTp doses for malaria based on data collected from several districts in Tanzania in different periods (2004–2010) as documented by other reporters

***Year of survey***	***Survey team/Organization***	***% of clients who received IPTp-1 (1***^***st ***^ ***Dose)***	***% of clients who received IPTp-2 (2***^***nd ***^ ***Dose)***
2004-2005	TDHS	53	22
2007-2008	NATNET	50	45
2007-2008	THMIS	57	30
2010	TDHS	61	26

*Source*: NMCP, USAID-MoHSW-MAISHA Project [38].

**Table 4:** Estimated percentage of uptake of IPTp-2 among the pregnant women identified at HFs in Tanzania as documented by other reporters

***Health facility level***	***Rate of IPTp-2 uptake***	***% HFs reporting SP stock-outs***
Hospital	46%	9/19 (47%)
Health Center	27%	7/11 (63%)
Dispensary	66%	3/10 (30%)

*Source*: NMCP, USAID-MoHSW-MAISHA Project [38].

### Ethical considerations

The ethical clearance certificate for this study was granted by the MoHSW through the Medical Research Coordinating Committee. Contacts were also made to regional and district level authorities representing central and local governments before and after reception of the national ethical clearance certificate. The management authorities of the selected HFs as well as the frontline HWs were also contacted about the planned study. The study population signed an informed consent form for their voluntary acceptance to participate in the study and for their approval of the study findings to be disseminated widely for policy, research and public use, particularly for the information that were not considered to be confidential. Other details on the informed consent process are documented elsewhere [18,22,31-33].

## Results

### Quantitative findings

#### Data actually documented and their quality

The data presented in the first two tables (Tables 1 and 2) for both districts do provide a general picture on numbers of ANC clients who were registered to have received the two doses of IPTp ( IPTp-1 and IPTp-2) during the specified years in this case. Also the abbreviation IPT appearing in Figure 1 represents IPTp since the commonly used abbreviations in most of the national official documents are IPTp-1 for the first dose given and IPTp-2 for the second doses [36,38].

These tables illustrate that the number (proportion) of the IPTp-2 recipients was far less than those of the IPTp-1 recipients, a situation that seemed to be common in other districts in the country [39].

The lack of proper systematic documentation of all the data in MTUHA at HF level as discussed above made it practically impossible for the data collectors to trace and count the same individual women who actually received two or more doses during their current pregnancy. This means, not all the women who are marked to have received IPTp-2 are the same individuals as those who received IPTp-1. As revealed by the HWs, this happened so due to the tendency of some women to contact different HFs during same pregnancy period as and the irregularities in their ANC clinic attendance behaviours. That is, one might receive IPTp-1 at clinic ‘X’ and IPTp-2 at clinic ‘Y’. The time allocated for study could not allow the data collectors to continue sorting out by names the clients who contacted specific facilities where they actually did or did not receive IPTp. Participants claimed that the gaps identified in the HF based data from MTUHA books in relation to actual IPTp coverage would have been filled if supplementary data collected from other sources e.g. community sentinel surveys were sought to observe the records shown on the current and past pregnant women’s ANC cards. This was not done under the current study due to time shortage as well as an overlook done at the study design stage.

There seemed to be unsystematic variations in the number of clients who received IPTp doses in different reporting periods – months, quarters and years. In most of the available literature on coverage of women receiving IPTp at HF levels within SSA, data are reported on annual basis rather than on monthly or quarterly basis. The present study came up with a little different view from this traditional approach by indicating the variations in the number of pregnant women covered with IPTp doses on quarterly basis after getting the data for each month. The observed differences in numbers of the clients covered could be mainly related to (or reflected by) seasonal variations in the ANC attendance rates/behaviors. One of the factors possibly influencing such behaviours include the women’s low access to cash money or some individuals being more occupied with domestic activities such as farming rather than being attributed to systemic constraints such as drug supply or staff performance differences. While the latter point of view was given by several CHMT members and NMCP officers, more systematic survey involving interviews with community members would be more appropriate to confirm how this was true or untrue.

As shown by data from Mkuranga, in 2004 the number of the pregnant women recorded to have received IPTp-2 had increased between the first and second quarters. However, there was somehow a steady decline during the third and fourth quarters. In the following two years, the clients registered for IPTp-2 had decreased by comparing the early months and the later months in the respective years. Despite the number of IPTp-2 recipients during the third and fourth quarters of 2004 indicating to be high, the actual coverage in terms of proportion of pregnant women receiving IPTp-2 is unknown due to lack of reliable denominator data. It seemed that there should have been a representative population base as a reference to which the calculations could be performed, and as will be discussed later, there is an issue of whether to use HF data or a mixture of HF and community survey data to arrive at a realistic population base as a reference when attempting to determine the realistic denominator. Thus, the presumption that coverage in both districts was still less than the desired level of at least 80% as per national target [18] could not be supported by the patchy data available at least for some years. Forcing the calculations by using the data for the other years to convert the number of women receiving doses to percentage coverage could not just be possible, but also could make no sense. The reliable conclusion might not be made justifiably in the absence of catchment population data indicating actual HF/service utilization despite general evidence from most SSA countries indicating levels of coverage of IPTp recipients as still being only modest [1,40].

Up to the end of the survey under the present study, records for the last quarter of year 2006 were not yet available in MTUHA books reviewed in Mkuranga. In Mufindi, the books indicating such data could not be traced at all. As reported by the HWs, such documents were picked by the CHMT members during their supervisory visits on reason the documents had to be used at district level for planning and higher reporting purposes, but were delayed to be brought back until the present study was being implemented. Also, such documents could not be traced at the district level when the study team decided to make follow up. In Mufindi also, the number of women registered to have received IPTp-2 increased between January and December in 2004 and 2005, and between March and December in 2003. Frontline HWs and CHMT members from both districts linked the decline in the number of IPTp-2 recipients during the second quarter of year 2003 with the period when the MoH withdrew the stock of defective anti-malarial drugs containing sulphur at all HFs throughout the country before replacing it with a new supply of anti-malarial drugs. This was confirmed by the NMCP officers and other authors [17,18,34]. These reporters regret the reports given about temporary stock-out of SP at several HFs in a number of districts in Tanzania. Other factors were related to women’s ANC seeking behaviors and HWs’ occasional no-adherence to national guidelines for IPTp administration, including proper record keeping.

More could not be known to be discussed or argued well in terms of the patterns of the data on the number or proportion of the IPTp recipients per districts because of lack of a reliable denominator that could be used as the basis for making comparison and if this were done, it could mainly be based on speculations. However, a mere comparison of the crude data presented in Tables 1 and 2 indicates roughly that the Mufindi district had recorded a higher number of women who received IPTp-2 for the respective quarters than Mkuranga district while the opposite is true for some years. This has been talked more about later under the discussion section based on the speculations of the present paper’s first author. In addition, the results obtained from observations on the ANC cards of the clients who were found at HFs by the present study team indicated that 50.3% out of the 210 women who were found in Mkuranga and 49.3% out of 199 of their counterparts found in Mufindi had received the third dose of IPTp (IPTp-3). The system of administration and recording of IPTp-3 was not uniform at all the study HFs. District Medical Officers, the reproductive and child health coordinators as well as some frontline HWs in both district gave their opinions about this based on their experience with working in other districts. They claimed that the observed variations in the system of data recording at HF level were actually a nationwide problem. Also all stakeholders interviewed from HF levels through to national level were of the perception that the system of administering three doses of IPTp was unofficial as the national guidelines emphasized administration/delivery of two doses, each to be administered in the specified pregnancy gestational ages, therefore, the practice somehow contravened the existing guidelines. As commented by the frontline HWs and some CHMT members interviewed, it was one non-governmental agency operating that organized seminars with frontline HWs and some health managers at district level to orient and directed the frontline HWs to administer IPTp-3.

However, the NMCP officers during the follow up interview acknowledged the latest WHO guidelines clearing the doubts by specifying that more than two doses could be administered [11]. So admitting this NGO as having been correct, the officers commenting argued that it was realistic since there are pregnant women who practically register for their first booking at the clinic very early during the first trimester of their pregnancies and consistently continue attending clinic as scheduled by the clinic staff. Therefore, waiting for these clients to receive IPTp until their pregnancies reach 20 weeks is partly to deny their right to get protection early. It was added that even the statement given in the guidelines is that ‘administer at least two doses’ and this means that more than three doses could be administered if conditions allow, so there was no reason to judge the NGO trainers wrong. Earlier, even the national level officers perceived that administering more than two doses was contrary to policy guidelines from the MoHSW [18].

### Different methods of estimating/measuring IPTp coverage

Discussing about the data from one of the recent surveys undertaken by the MoHSW in collaboration with several agencies [38], one of the national level officers interviewed in the present study was concerned with the nature of the survey designs, sampling strategies and areas covered by the surveys. An example was given about the three different organizations that recently came up with different figures indicating the proportion of the ANC clients covered by IPTp doses (abbreviated alternatively as IPT in this case) in selected districts in Tanzania in different years (Figure 1).

Such officers also questioned about the estimated coverage rates of the IPTp dose recipients measured by levels of HFs as indicated by the results from other surveys in the country (Tables 3 and 4). Doubt was expressed regarding the denominator used for arriving at the calculated figures, albeit it was not specified to guide the readers make fair judgment on the data presented. It was only presumed by the officers approached at NMCP level that most likely the national and international standards have been used whereby IPTp coverage is determined as a percentage of all pregnant women registered at ANC clinics during the specified period [7]. Interestingly, one officer gave reference to some critics who still criticized some methodological approaches employed to arrive at IPTp coverage estimations by comparing HF and community survey data [5,17].

The data presented in the foregoing tables were collected by the stakeholders from the MoHSW especially from the NMCP WHO- Country Office [35,39], other partners to the MoHSW including United States Agency for International Development (USAID), as well as other private sector agencies from both within and outside Tanzania [39]. As argued, knowing the denominators used to arrive at the coverage data enables the readers to compare the data obtained from present study districts and other sources. It was added that the indicated IPTp coverage rates seem to represent the percentage of all pregnant women who received the doses out of those who attended ANC clinics during the specified period, thus the data presented are HF based. Need for community survey data was mentioned as being necessary for comparing with the HF based data.

The national officers commented that in analyzing the issue of IPTp coverage, it is important for the analysts to look at the issue broadly through analysis of systemic challenges/opportunities related to the supply of essential drugs (e.g. SP) at HF levels, how clear the IPTp guidelines are, and human resource numbers, skills and motivation on one hand, as well as personal or traditional issues such as pregnant women’s ANC seeking behaviors, attitudes towards SP (e.g. for IPTp) on the other hand. It was claimed by the national officers that the IPTp data are sometimes collected in different years/periods and that even when they are collected in the same period by different teams they tend to differ considerably. This raises the questions regarding data that are correct to be relied upon, the critical issue being the methods used in their collection including selection of the HFs by number, types, and location, as well as the timing of the surveys and personnel involved in the survey, particularly in data collection.

Although there is no explicit explanation and criteria for such feelings, there seems to be some indication of the monthly variations in the attendance of pregnant women who were eligible to receive IPTp in each district, apart from the issue of SP stock-outs at HF levels as explained below based on the key informant interviews made in the present study and as illustrated by the data presented (Figure 1).

#### Stock out of SP for IPTp

The issue of occasional stock out of SP at HF levels was not the only and main reason for the observed variations in the coverage of pregnant women receiving IPTp. Occasionally, pregnant women were being advised to procure SP from retail sources for IPTp purposes when there was no SP to offer for free. This issue featured to one of the important themes during the interviews at HF levels [32], district level [22] and national level [18]. Also, this issue seemed to recur or become chronic given the reports documented elsewhere about its happening [3]. As shown in Figure 1 above, coverage of IPTp-2 (abbreviated as IPT2) beneficiaries rose from 18% in the first quarter of 2007 up to 69% in the first quarter of 2008 after which it declined up to 32% in the first quarter of 2009 before it began to rise again albeit at a low rate than it did between the first quarter of 2007 and that of 2008. Different views can be given about these variations, but in general, the IPTp-2 coverage rate had remained lower than the IPTp-1 coverage rate between 2007 and 2010. There seems to have been an inverse relationship between stock-outs days and IPTp coverage since the less the number of stock-out days the higher the rate o IPT coverage, as one would have expected.

### Qualitative findings

This section covers reasons as to why the quantitative data for the periods reported above were so poor. Such data also gives a reflection on what the key informants and the present authors perceived to be with regard to the implications the incomplete or controversial data might have had on the ability of the study districts and MoHSW to plan for the IPTp related interventions.

#### The recommended system of data/record keeping and the actual practice

These MTUHA registers are standard and recommended to be used systematically in both the public/government and private HFs. The documents normally used for keeping IPTp data along with the records taking of the data for other RCH services include MTUHA Book 6 version 2.0 (but sometimes in MTUHA Book 2). Frontline HWs are also required to record the client’s personal RCH related information on her ANC card. However, in the event whereby the cards are out of stock at HF level, the HWs are allowed to use ordinary exercise books either supplied to the clients or procured by the clients themselves. The personnel responsible for keeping the ANC (including IPTp) data are those working at the RCH clinics, and the data collected at the clinic level are then reported at district level, particularly to the district CHMTs/DMO’s Office. The CHMT is comprised of the health managers responsible for the planning, supervising the implementation, and execution of the health services in the district/council areas [22]. The data compiled by CHMTs from all HFs in the district/council area of administration are usually sent at central (national) level, particularly to the NMCP. Among the CHMT members is the district nursing officer (DNO) who acts as the overall in-charge of nursing services in the district. There is also a district reproductive and child health coordinator (DRCH-Co) who works on malaria related issues closely with the district malaria and integrated management of childhood illnesses focal persons (abbreviated as DMIMCI-Fp) and district health officer (DHO) (NMCP Managers, per comm.).

#### Un-standardized system of data/record keeping

According to claims from the HWs interviewed, observations made in the MTUHA documents at HF levels as well as the feedback obtained from CHMT members in both districts and national level managers [18,22], lack of a designated section/space for accommodating IPTp records has been forcing the HWs to use different systems of record-keeping. Inconsistency was noted in the way the records were being taken in relation to the IPTp services given. It was found that among the HWs found at same duty stations (clinics), some were using symbols like putting a tick in the open cells in MTUHA books against each client’s name marking those who have received IPTp doses; others just wrote ‘SP’ or ‘3 tabs’ or put a ‘Y’ (for Yes) in similar cells to indicate the actual delivery of the drug for IPTp. Also, there were some who put a dash (−) to illustrate that the client was not given the drug due to explained/unexplained reasons or just wrote ‘O/S’ to indicate SP as being out of stock for free delivery at HF level.

The frontline HWs from both districts acknowledged that seminars related to malaria were occasionally being organized by district health authorities involving CHMTs. The frontline HWs who were invited to such seminars were oriented on health management information record keeping issues, including those related to IPTp. Those who had never attended such seminars have been either oriented at their workplaces by their co-staff members who have had an opportunity to attend such seminars. They also occasionally get to be oriented by the district CHMT members during CHMT’s trips as part of their supervisory visits to HFs carried out on quarterly basis. Sometimes, the frontline HWs were using pencils to keep records in the MTUHA. This resulted into some records being either rubbed off through regular paper handling or completely lost after the pencil marks fainted. The remaining marks eventually became difficult to read after losing their original color. Some of the frontline HWs who were computer literate reported to be concerned with the lack of an electronic system for record-keeping at HF level. It was lamented that having such a system could have allowed the data/records to be reported from HFs to district or regional or national level directly, for example using the MS-Access program through the internet system. It was claimed that over-reliance on a manual system of taking notes on papers first in the registers and compiling the records for reporting the data to higher management levels later was cumbersome and unnecessary as it first of all increased the workload to the existing staff, and secondly it caused inconvenience to the officers sitting at higher levels to make follow up in their attempt to execute the records, including examining the correctness, completeness and consistency of the data reported from lower HF levels. These concerns were also shared by the district RCHCo and DMICI-Fp for each district, as well as by the core members of the district CHMT under leadership of DMO [22] and NMCP officers working at the NMCP Headquarters [18]. Apart from the issue of equipment shortage, particularly the budget constraints for purchasing computers for each of the HF-based personnel, it was argued by the in-charges of the study HFs as well as by the DMOs of both districts that another drawback was the lack of electric power at most of the peripheral HF. This means that even if the computers were made available for use, the power shortage could lead to their sub-optimal use, unless there were standby generators to fill the gap.

### Unclear and missing data

As mentioned before, missing the desired data at the study HFs was a challenge to the research team when it came to calculation of the percentages of the IPTp recipients for comparison purposes. The data expected in the MTUHA registers for the past years especially year 2002, that is, one year after the official introduction of the SP as the first line antimalarial drug and for IPTp as well as partly the data for the following year (2003) were not traceable (i.e. not indicated) in the registers at several HFs visited. The shortage of data for this period was more experienced in Mkuranga than in Mufindi. Even at the HFs where the documents for such years were found, the data for the IPTp doses administered during that period were not shown. Access to such data could help to calculate the percentage of the women who received IPTp out of the total number of the clients attended at the respective ANC clinics. That is, the actual data collected could give a misleading picture since they would give results that are not comparable with the results obtained elsewhere based on the national and international standards. Often, documents show that the IPTp coverage rate is calculated as a proportion of all pregnant women attended at ANC clinics during the specified period. This opinion was expressed by the officers at the NMCP level and DMO for each of the two study districts, and indeed at least some of the documents reviewed indicate so [3,15].

As claimed by one HW, the deficiency in the data required happened because the data concerned were sometimes not being recorded or documented. This refers to the period when majority of the frontline HWs were still unclear on whether or not they could make manipulation to squeeze the data/records taken from the clients into the meager spaces available in the MTUHA books. As an alternative, the HWs sometimes decided to use their own note-books for keeping records of the services they had delivered on each day with the plan of transferring such records in the appropriate MTUHA books later. For various reasons, the note-books could be eventually misplaced or lost. This happens, for example, during the process of shifting the documents from one office to another, particularly during HF renovation period. It could also happen when there was a relocation of the offices or the HWs with such data being transferred to other duty stations without leaving the records they have taken from their previous duty stations. The same records supposed to appear in the MTUHA registers had to be kept on the women’s ANC cards as officially recommended [21]. However, the HWs revealed that the IPTp data get lost when the ANC clients misplace their original cards or poorly handle their cards which eventually look either torn or with records too difficult to read after becoming dirty.

None of the reviewed MTUHA registers indicated the actual gestational age of pregnancy at which the women concerned received IPTp doses. It was only assumed based on the existing national guidelines that the recipients obtained the first dose between the week 20 (5^th^ month) and week 24 (6^th^ month) and the second one between week 28 (7^th^ month) and week 32 (8^th^ month) [21]. According to some of the district level officers and in-charges of the clinics, the indication of the actual pregnancy age at which IPTp was administered would have enabled the research team to assess HWs’ adherence to the guidelines for IPTp administration. The registers only indicated the clients who received IPTp before 20 weeks (<20 wks) or after 20 weeks (>20 wks) of their pregnancies, which are vague as they do not indicate the actual week of pregnancy. The point emphasized here is that even if it was officially and scientifically acceptable for the HWs to administer IPTp dose immediately after quickening, possibly some HWs could still administer IPTp earlier than 16^th^ week of pregnancy, wherefore, risking the dose recipients further which the MoH warned about [21].

Several HWs viewed that there is no way the current MTUHA could be manipulated to indicate the exact gestational age of pregnancy as it is indicated on the women’s ANC card. The inter-temporal observations made at three public HFs (one HC and two dispensaries) and one dispensary run by a FBO in Mkuranga as well as at one mobile clinic in Mufindi confirmed that HWs sometimes forgot to copy in the MTUHA books the same data as recorded on the clients’ personal cards. This is mainly due to the exhaustion of the frontline HWs who eventually become tired after attending a high number of the ANC clients and other patients who were overcrowding at the HF. This was due to the HF understaffing situation. An opinion was given that pregnant women’s ANC cards might provide more reliable records on IPTp doses administered as they contain information on parity, gestational age and IPTp coverage of both the first and second doses. Nevertheless, this could be possible had sentinel site recording of data from ANC cards been done, but unfortunately it has not been the case.

#### System of recording, computing and reporting IPTp coverage data at various levels

As reported by the national level officers and their assistant managers at district level, the data recorded at HF levels are intended to, and usually, compiled to be reported at district level. The reason given is that such kind of reporting helps to pool together the data which are ultimately used by the CHMT to plan evaluation of the performance or achievements by comparing the current situation with the initially planned activities during the respective year. This could also act as the basis for better planning to improve the performance in the next reporting period. The data compiled by CHMTs have traditionally been used for reporting to higher (e.g. national level) authorities, and this is done on quarterly basis and annual basis along with other health service data. Thus, CHMTs have responsibility of ensuring that the data gathered are properly documented at the points of their collection (e.g. at HF level) before being transferred to higher levels. In practice, this is not always the case, as reports from the HWs indicated, and as confirmed by a review of records on CHMT supervisory schedules/plans. The national guidelines specified that at least one visit be carried out by the CHMT for each HF per quarter. It happens, however, for a number of HFs sometimes facing the problem of not being visited for supervision by the superiors from district CHMT or other levels. Failure to carry out some supervisory visits is caused by either the lack of means of transport by the respective supervisors or other official commitments within or outside of the districts. In addition, it was lamented that visiting each HF for supervision as planned by the CHMT could offer some technical guidance/support to the frontline HWs on matters related to recording the HMIS data into MTUHA. From Mkuranga, reports from both frontline HWs and district managers that were confirmed by the team involved in the present study revealed that the shortage of means of transport or inconvenience caused by some interruptive events increased the work-pressure of the HWs and this disturbed such providers further to perform their originally planned health service activities. The district CHMTs and national level respondents appreciated this to be a barrier in the first place, and understood that this combined with inability of some of the HWs to interpret the national IPTp guidelines and complying with them in the administration of IPTp doses eventually affect the data collection (record taking) at HF level and data reporting system at district level and then to the national levels [21].

#### Effects of human resource shortages and drug stock-outs on IPTp data reporting

The stock-out of SP for delivery free of charge to all ANC clients was identified by the district and national level respondents as one of the stumbling blocks to the efforts aimed by the government toward universal coverage of IPTp services. This is challenge especially when combined with other reported barriers to the attainment of the national IPTp target. As an example, a comment was made that the issue of lack of appropriate supportive supervision was a crucial concern calling for a concerted intervention. Apart from helping the HWs in their duties by giving them some kind of the on-the job training, the supervisors could receive information about issues related to drug stock-outs and possibly address them accordingly [18,22]. The HW shortages seriously facing most of the peripheral (and public) HFs were mentioned by all of the key informants at all levels (HF, district and national) as a problem requiring urgent and more concerted measures or attention to be paid to if the HMIS and delivery of quality services have to be realized in the health care system. Having clear guidelines in place where there is acute shortage of personnel to use them and yet expecting quality services and data records was perceived to be a nightmare.

Meanwhile, reports were obtained about occasional failures of the routine HWs to reach the far remote or difficult to reach peripheral settings for delivering RCH services. Due to scarcity of staff, the few HWs available occasionally fail to keep records for all the pregnant women who have been attended including those receiving IPTp doses at outreach clinics due to high work-pressure/load. This situation was reported in both study districts. The reporters and their superiors at district and national level shared the view that without deliberately planned and enforced strategy to give basic support, it would be unfair to judge the frontline HWs as poorly- or non- performing in data management.

## Discussion

### The observed deficiencies in the data recording and reporting system

The findings from the present study reveal the chronic flaws in the data documentation at FF levels, and this includes the missing of the data, erroneous recording of the data in the HMIS registers, and even reporting such data. The implication of this is that the use of the reported data for managerial decision-making particularly at the planning stage for IPTp interventions at the district level as well as at NMCP/national level is consequently questionable. As perceived by other authors (cited in the background section of the present paper), the improper clients’ record taking at HF level opens chances for the HWs to guess by filling the unreal data in the MTUHA registers and in other primary health care reports usually used by district and national level health authorities for arriving at various health service management decisions. That is to say that the chances for under-reporting or over-reporting of the services either actually delivered or required to be given are open. Therefore it would make sense to collect and report the data that could help the higher level decision making authorities to make better informed decisions. This is possible if such authorities deliberately work hand in hand with statisticians and other data experts to address the existing HMIS data deficiencies including measures improving the current HMIS/MTUHA format and utilization [26]. Interestingly, WHO and her allied organizations/authorities continue appreciating that adequately informed policy or managerial decisions need to be guided by reliable, timely, and well packaged evidence. This includes among other things having in place a proper data recording and reporting system at all levels [1]. As detailed in the present paper’s background section given above, the present study findings provide additional and useful evidence supporting the facts documented before by other authors about the issue of HF-based data limitations caused by the existing deficiencies in the national HMIS of Tanzania. Such findings also show the existing differences in the administration of IPTp doses including the timing of delivering the scheduled doses either among the staff working at the same HF level or between staff working in one area and those working in other areas. The contributing factors for the observed differences may partly include the influence of ANC attendance behavior of the pregnant women contributed by a number of factors - some being social, cultural, economic, and health system (systemic) on one hand. On the other hand knowledge and perceptions of the service providers in relation to IPT may contribute and this includes the timing of the doses and the possible benefits or risks of using SP for IPTp [4,5,15,17,34,36]. There is a general shortage of evidence on the factors leading to the observed low-to-moderate coverage of IPTp services in SSA despite records on high ANC attendances [3], and the issue of dilemma about existing guidelines for IPTp administration, shortage of facilities including medicines and record keeping facilities and other inconveniences facing the frontline HWs responsible for delivering the services continue being pointed out among other challenges [41].

Apart from the shortages or weaknesses inherent in the documents e.g. register books that are in place for record keeping, the role of inadequate morale of the frontline HWs to keep the data cannot be underrated. There is need for finding out more practical ways of training and motivating the HWs on better techniques for collecting and reporting reliable data on various, health service activities malaria IPTp being one of them. This point is validated by the HWs responding to this study who complained about limited training opportunities on focused ANC issues. Thanks to the MoHSW which through its NMCP has acknowledged this issue of HWs training on focused ANC including MiP aspects as an essential means through which the quality of services delivered and the data recorded could be improved [42]. The need for using the sentinel site data involving both HMIS records and records taken directly from the women’s ANC cards as viewed by several district and national level officers is also important to take into account. Apart from malaria, there are various health problems needing proper data documentation and in the situation whereby there is a serious shortage of HWs, it is likely that data recording mistakes or deficiencies will remain. Therefore, the authorities concerned should look at how better the HMIS data pertaining to different diseases/services can be dealt with at HF levels. The measures to take may include those looking at how the various data from different sources could be integrated and properly managed at the point of collection without confusing or discouraging or inconveniencing the frontline HWs unnecessarily. As warned by other authors, it is not a matter of emphasizing on data integration without examining on the ways of ensuring supportive or mutual working relations which support the health information system at all levels - global, national and local (e.g. HF) [26].

Also, the gaps depicted in the present study about lack of special sections for keeping data on the IPTp doses delivered to the ANC clients are supported by reports from previous studies. Such studies found a general lack of properly designed routine data collection, collation, analysis, and reporting systems in Tanzania. Meanwhile, different surveys indicated different coverage rates of both the IPTp-1 and IPTp-2 recipients [17,34,36,39]. The issue of which is the best denominator based on which estimation/calculation of IPTp coverage rates could be performed is likely to continue raising debate if there is not better means for appropriate data to be collected through the routine HMIS. Meanwhile, having data on gestation age of the clients who were given IPTp to be better reflected in the MTUHA/HMIS registers is also important rather than leaving this to be shown on the client’s ANC card or requiring one to go deeply into MTUHA Book 2. The gestational age data would be very useful for monitoring the correct implementation of IPTp and evaluating the outcomes of SP among the users. Of course, a crude measure of IPTp determined based on the records taken in the HMIS or on the clients’ ANC cards is still subject to debate: having proper records does not necessarily represent actual use of the intervention, and as other experts recently noted, evaluation of specific interventions like IPTp is still subject to debate since the analysts have to ensure that they control for other variables such as use of ITNs and other preventive products/measures [6].

In order to realize acknowledgement from the frontline HWs and communities about the efforts made, the officers working within the NMCP in collaboration with those in the Department responsible for planning and policy at the MoHSW of Tanzania have to continue supporting and when possible participating in periodic surveys as part of the strategies towards improving the HMIS/MTUHA system. The surveys should include among other things continuous monitoring and evaluation of the applicability of the revised national MTUHA system and how any of the gaps found in this system can be supplemented with data collected using other means and sources. There is also need for setting standard and user-friendly data keeping guidelines to ensure easy implementation by the frontline HWs who should be well trained, motivated and regularly supervised on health service provision aspects [17,19,22], as suggested by other observers on HMIS in general [1,23].

### How could HMIS/MTUHA system be modified to support IPTp data documentation?

The MTUHA system designers could learn from other vertical or integrated programs such as programs on sexually transmitted illnesses, Tuberculosis and Leprosy, and Expanded Program for Immunization (EPI) and even HIV/AIDS. These systems/programs have managed to use a unique system of data recording often summarized and integrated in the MTUHA for reporting purposes. The lessons to learn from these systems may include an introduction of new or revised registers for accommodating routine data from new interventional strategies such as IPTp as are usually collected for parallel vertical programs such as those mentioned above. Thus, the current MTUHA registers may be revised by inserting tables with spaces for recording actual gestational ages of the pregnancies and the doses administered for IPTp for each client who is attended by the service provider. This is possible since similar kinds of records are usually shown in the ANC cards. Involving the key stakeholders including people with expertise in health informatics or health management information systems as well as frontline HWs whose experience from their day-to-day service practice matters would make an important contribution. The advantage of adding additional tables or cells for recording the gestational ages of the clients receiving the IPTp doses is justified by the fact that sometimes pregnant women misplace their ANC cards when they revisit the clinics. This means, the HWs confronted by the women who have lost their ANC cards may face troubles to determine the actual age of a client’s pregnancy and may miss other important information that is required to guide the staff as reference for deciding to administer or waive administering particular services (IPTp doses may be inclusive). Of course, even with remarkable improvements, a perfectly designed HMIS register that can accommodate all the relevant data needed may not be realized given the fact that information needs may change as new health intervention or programs come into existence.

### Dealing with stock outs of SP at health facility level for IPTp purpose

The varying rates of IPTp coverage usually indicated by survey reports cannot be discussed without one having to look at the issue of drug supply at service delivery points and unpredictable trend of patients/clients ANC seeking behaviour that may change from time to time. If the attendances exceed the supply of the drugs available in stock at HF level, it is obvious that some of the clients will end up not getting the desired medicines. Without looking at better practical ways of ensuring adequate supply of medicines at HF level all the time, the patients and frontline HWs will continue being dissatisfied with the drug shortage challenges/difficulties they face. The occasional drug shortages at several HFs is a chronic problem and not for IPTp-SP alone, and is mainly due to the systemic weaknesses in the drug distribution logistics in Tanzania. Thus, as patients and service providers face inconveniences, they get demoralized, and this has negative effects on the delivery and utilization of the intended/desired services [32,33] and ANC clients in the present study districts. Similar challenges were found in other districts, as revealed by the reviewed data (Tables 3 and 4, Figure 1) and other reports [15,34]. At least on paper (theoretically), the MoHSW acknowledges this fact by calling for measures to be taken to ensure the availability of sufficient SP at all HF levels [42].

### Strengths and limitations of the present study

The data usually reported in the international literature about the coverage rates of IPTp recipients do not illustrate monthly variations in the coverage. Often, the data reported are usually aggregated for annual reporting basis. The present study helps to at least reveal some of the monthly variations, for instance, the monthly and quarterly patterns in the number of women who received one dose or more doses of IPTp-SP. The immediate challenge to these data is that the study did not cover more questions to confirm/establish what might have been all the key causes of the observed variations. Instead, some of the opinions expressed herein from the key informants or the present authors are based on speculations. Another weakness of the present study is that there is limited comparability of the data presented because of the observed differences in, and a few numbers of, the HFs covered, the reference years for which the data presented belong, and lack of a reliable denominator. In the case of a denominator, it is suggested that the number of women registering for ANC could be appropriate [7]. However, in the latter denominator case care should be taken by the data recorders to avoid double counting the clients who register with slightly different spellings of the same name as well those visiting more than one clinic in the same pregnancy period. It remains unclear, therefore, whether or not the remaining proportions of the clients recorded to have received only IPTp-1 at the study HFs in the two districts were able to receive IPTp-2 elsewhere as the authors acknowledge that this has not been studied. It would have been better if the HF data were compared to the data collected through community surveys of the pregnant women who received IPTp in the most recent period. Data from community survey is often recommended for various forms of health services targeting people from household level [30], and this applies even for the ANC services. Furthermore, the study would provide important information for both knowledge and policy improvement if it showed the coverage of IPTp over the years looking at the time of first booking, time for IPTp-1 and IPTp-2 and relating these variables with the demo/ethnographic factors. Finally, this study pressed emphasis or focus on determining/reporting the number of pregnant women who received the first and second doses of IPTp, with little attention paid to the recipients of IPTp-3. Nevertheless, the advantage of administering three doses is scientifically reported to be minimal for HIV-negative clients and this has stimulated debate among physicians and other scientists before WHO gave a resolution that now IPTp doses can be administered more than twice in the recommended gestational age so long as there is a one month interval [11]. This is something to look at more carefully by those who take responsibility to design HMIS registers or ANC cards whereby more than 2 doses of IPTp-SP can be administered. According to WHO, even in the absence of resistance to SP, HIV positive women require more doses of SP to achieve effective protection against malaria in pregnancy than women who are HIV negative [11]. In the document where the latter WHO’s recommendation is placed, it is also revealed that the results of an unpublished meta-analysis comparing 3 or more doses of IPTp-SP (median of 4 doses) with the standard 2 dose-regimen in 7 randomized trials demonstrated the benefit of more doses; meanwhile preliminary results of ongoing monitoring studies of IPTp-SP effectiveness suggest that IPTp-SP effectiveness could be improved with the administration of a 3-dose regimen. A final weakness of the present study is that it did not investigate the knowledge and practice of the frontline HWs regarding delivery of contrimoxazole to HIV positive pregnant women instead of SP. This category of clients might be among the group of those who were not given SP for good reasons in the present study districts. Therefore, the question is whether to treat this group of women as having a ‘missed opportunity’ for IPTp instead of treating their information as missing data in the HMIS registers. WHO warns that SP should not be given to HIV positive clients since doing so might result into contraindications [11].

## Conclusion

The present study makes it clear that a strong HMIS is crucial for successful planning for the services desired in the country and this fact applies to whether the information is related to the epidemiological data for particular diseases or for services. According to information communication and technology experts, reliable and timely health information is one of the foundations of effective health service management and public health action, and while most countries have been using plans with indicators and targets to monitor progress and performance, their ability to do so has been hampered by availability and quality data; and in most countries, HF reporting systems continue to be plaughed by data quality problems [1]. In light of these facts, the findings from the present study in Mkuranga and Mufindi suggests that a better recording and reporting system on the coverage rates of the IPTp recipients could only be realized if the frontline HWs received the necessary support. This includes service providers receiving adequate supply of quality SP, regular HW training through seminar-sessions or through routine supportive supervision at HF level on issues related to the IPTp administration and HMIS that should go hand in hand with promotion of early booking and consistent women’s ANC attendance and establishment of user-friendly health management information system. Other measures include careful and sufficient deployment of the existing HWs and recruitment of new ones to fill the existing gaps as much as national or district budget allows. This may help to ensure there is a fair balance between number of the clients attended over time and the staff manning levels at different rural and urban HFs. Some of the deficiencies reported from the present study in relation to data gaps do not necessarily require national intervention - they could be addressed by district level authorities. Looking at means for integrating HF-based data and community survey data could help to give a more representative reflective picture on health services. The design for integration of health service data need to be carefully done in manner that does reduce the inconveniences currently facing the frontline HWs in terms of data/record taking and reporting in instead of increasing such inconveniences. Also, the data collectors at HF level should be motivated to get the right sense of why they should bother to take records carefully and this includes among other things making them appreciate the use of the data being collected right from the point of data collection (HF level) to higher levels. However, some of the identified shortcomings relate to the broader concerns addressed in the main study that health systems capacity is limited [31] and this has also been documented in other papers [18,22,32]. Therefore, additional service workload including that of data management cannot be counted on within existing scarce resources. There must be explicit choices including either to decrease other efforts to a more generally less appropriate approach or to specify additional resources for strengthening and sustaining a more separate or more vertical approach that can make the health information system function more effectively. Thus, a general HMIS strengthening may solve the data problem, but only by competent and motivated personnel being available at facility level. Unfortunately, this is a hardly priority in relation to needs for service provision in Tanzania and many other resource poor countries. A careful integrated and contextual systems planning cannot ignore or be bypassed by any new program. Any concerted measure to reduce or eliminate the imbalance between the increasing demand for indicators and reporting and the actual efforts to strengthen country health information systems and its core sources would be highly commended since this is still a major problem of the majority of low and (lower) middle countries [1].

## Abbreviations

ANC: Antenatal care; CHMT: Council Health Management Team; DHS: Demographic and Health Survey; HMIS: Health Management Information System; HWs: Health care workers; IPTp: Intermittent preventive/presumptive treatment during pregnancy; MoHSW: Minsitry of Health and Social Welfare; SP: Suphadoxine-pyrimethamine; MTUHA: Mfumo wa Taarifa za Uendeshaji wa Huduma za Afya – Tanzania (HMIS-Tanzania.); NATNETS: National Insecticide Treated Nets Program (of Tanzania); NATNETS: National Insecticide-treated Nets Strategy; NMCP: National Malaria Control Programme; RCH: Reproductive and Child Health; RCHCo: Reproductive and Child Health Coordinator; TDHS: Tanzania Demographic and Health Survey; THMIS: Tanzania Health Management Information System; USAID: United States Agency for International Development.

## Competing interests

All authors worked on this paper, approved it, and then declare no competing interests.

## Authors’ contributions

The listed co-authors commented on the paper and GMM’s research proposal for his PhD training. The four ICB, PB, PM and JB cooperated in supervising GMM’s PhD study work under the leadership of ICB and PB. GMM conceived the study as a PhD student by then, implemented and defended it for his PhD graduation in January 2010; conceived and wrote the first and final versions of this manuscript. Additional final round comments were given by Drs. MM, FM, LEGM, JNI and mainly PB. All authors read and approved the final manuscript.
